# Teen Perspectives on Integrating Digital Mental Health Programs for Teens Into Public Libraries (“I Was Always at the Library”): Qualitative Interview Study

**DOI:** 10.2196/67454

**Published:** 2025-03-13

**Authors:** Ashley A Knapp, Katherine Cohen, Kaylee P Kruzan, Rachel Kornfield, Miguel Herrera, Aderonke B Pederson, Sydney Lee, Kathryn Macapagal, Chantelle A Roulston, Kaleigh Clarke, Clarisa Wijaya, Robert Simmons, Latonia Jackson, Simrandeep Kour, Sandra Franco, David C Mohr

**Affiliations:** 1 Department of Psychiatry and Behavioral Sciences Feinberg School of Medicine Northwestern University Chicago, IL United States; 2 Department of Medical Social Sciences Feinberg School of Medicine Northwestern University Chicago, IL United States; 3 Department of Preventive Medicine Feinberg School of Medicine Northwestern University Chicago, IL United States; 4 Department of Psychiatry Harvard Medical School Massachusetts General Hospital Boston, MA United States; 5 Bradley University Peoria, IL United States; 6 Department of Psychology and Psychiatry University of Illinois Chicago Chicago, IL United States; 7 Oak Park Public Library Oak Park, IL United States; 8 Chitkara University Chandigarh India; 9 Chicago School of Education Loyola University Chicago, IL United States

**Keywords:** public libraries, digital mental health, teens, youth, adolescents, anxiety, mental health, implementation, safe spaces, mobile phone, smartphone

## Abstract

**Background:**

Rising rates of anxiety among teens necessitate innovative approaches for implementing evidence-based mental health support. Public libraries, seen as safe spaces for patrons with marginalized identities, offer free public services such as broadband internet access. Many teens spend significant amounts of time in their local libraries due to the safety of this space as well as the trusted adults working there. The American Library Association has shifted its priorities to focus more on mental health through employing social workers and providing mental health programs. As such, public libraries may be promising sites for the implementation of digital mental health (DMH) programs for teens.

**Objective:**

This study aimed to examine how teens who attended their local public library experienced and managed their anxiety, what mental health supports they were interested in receiving, and how DMH programs and public libraries can meet their needs.

**Methods:**

We interviewed 16 teens aged 12-18 (mean 15.2, SD 2.0) years who used the library frequently at the time of the interviews. Of these teen patrons, 56% (9/16) identified as female, 31% (5/16) identified as male, and 12% (2/16) identified as nonbinary. Most (11/16, 69%) identified as either White or Black or African American individuals, with the remainder (5/16, 31%) identifying as Hispanic or Latino or Chinese American individuals or with ≥2 races. The interviews were individual and semistructured, designed to elicit recommendations for designing and implementing digital tools in libraries to improve teen mental health. Interview transcripts were coded by multiple coders using thematic analysis to synthesize key themes.

**Results:**

Teens reported experiencing uncontrollability, unpredictability, and anger related to their anxiety, which they managed using strategies such as guided breathing, distress tolerance, and social connection. They also talked about other helpful management techniques (eg, progressive muscle relaxation, journaling, and mood tracking). Teens underscored the importance of pairing mood tracking with daily activities to reveal patterns. They also stressed the significance of context and anxiety severity when choosing anxiety management strategies. Teens underscored the centrality of the public library in their lives and their view of it as a safe space where they can easily access resources and connect with friends and trusted adults. When considering the design of a DMH program implemented in libraries, they suggested including personalization for different identities, gamification, and simple navigation. Teens emphasized the importance of protecting their privacy within digital programs and that their end goal was to use the skills learned in the DMH program *offline*.

**Conclusions:**

Teens who frequently used their local public library expressed interest in receiving digital tools via libraries to help them manage anxiety. Their recommendations will help inform future research on the adaptation and implementation of DMH programs for teens in public libraries.

## Introduction

### Background

Researchers, clinicians, and policy makers in the United States have declared a teen mental health crisis due to increasing rates of internalizing distress combined with limited access to mental health programs [1-3]. The Centers for Disease Control and Prevention found that over one-third of teens in 2019 experienced persistent feelings of hopelessness, a transdiagnostic predictor of anxiety and depression [4,5]. Anxiety is particularly common among teens, with rates increasing roughly 27% between 2016 and 2019 [6] and continuing to rise since the pandemic [7]. Prevention and treatment efforts for teens have the potential to reduce the likelihood that internalizing disorders will persist or worsen in adulthood [8], but demand far exceeds the available supply of teen mental health programs [9,10]. Increasing the number of providers would not fully ameliorate the issue as many teens face additional barriers such as high treatment costs, lack of transportation to clinics, and complex regulations regarding parental consent [11]. Consequently, advocates call for researchers and clinicians to meet teens where they are by implementing mental health interventions in settings frequently used by teens [12-14]. In this paper, we focus on public libraries as a promising yet understudied setting for delivering teen mental health programs, particularly programs targeting anxiety.

Many teens, especially those with marginalized identities, spend significant amounts of time in libraries [15]. Public libraries are a *third place*, where patrons can spend time without being expected to spend money [16,17]. The commercialization of space, particularly in urban areas, has led to libraries being one of the few indoor spaces teens can inhabit for free without facing accusations of *loitering*. As a result, libraries have transitioned in recent years to become beacons of public services, offering health services, professional development classes, art workshops, and more [18]. An increasing number of libraries now offer teen-specific programming [19-21]. Libraries are also seen as a *safe space* for teens with marginalized identities, including historically underserved racial and ethnic minority (HURE) teens; teens who identify as lesbian, gay, bisexual, transgender, queer, intersex, or asexual (LGBTQIA+); teens living in lower-resourced and disinvested areas; and teens living with mental health conditions [15,16,18,22-27]. Unsurprisingly, the teen mental health crisis is visible in public libraries. Within the last decade, the American Library Association has shifted its priorities to focus more on mental health through actions such as employing social workers within public libraries and providing patrons with mental health programs [25,26,28]. At the same time, librarians report wanting more mental health support for teen patrons that is accessible and on demand and requires little additional training for already overtaxed staff [15]. Accordingly, interventions that leverage digital technologies, or digital mental health (DMH) programs, may be well suited for use in libraries.

DMH programs are an ideal way to reach teens for several reasons. Teens commonly use the internet for mental health support and psychoeducation [29]. DMH programs can be implemented in practically any location at relatively low cost. Libraries offer free broadband internet access and computer access to all patrons, allowing DMH programs to be easily accessed in this setting. Self-administered DMH programs provide availability on demand when teens need support, eliminating the need to involve a facilitator or caregiver and also satisfying the needs of teens for independence and autonomy [11]. Despite the promise of DMH programs, many face problems with user engagement and sustainment by organizations outside the research context [30,31]. Researchers have suggested that this may reflect, in part, that DMH programs are typically designed for research contexts rather than for implementation in real-world settings [32,33]. On average, it takes 17 years [34] for research evidence to reach real-world settings and change practice. These statistics have not significantly changed in decades, highlighting the need for innovative solutions to address this research-practice gap.

To ensure that DMH programs are implemented in real-world settings, researchers may look toward approaches that (1) center the community who will use the program, (2) design the program according to the community’s needs, and (3) evaluate implementation metrics outside of effectiveness. Community-based participatory research (CBPR) is an approach to research that prioritizes partnership and shared decision-making with community members impacted by the research [35]. The application of CBPR principles in the development of DMH programs ensures that DMH programs can positively impact the community of interest. Human-centered design (HCD) is defined as a set of methods that require that “the human perspective is considered from the initial conception to the eventual design, and that the people who will use and/or be affected by the designed product are involved in the process” [32]. Similarly to CBPR, HCD elevates the end users’ voices in intervention design and implementation, increasing the likelihood that design outputs will be acceptable and sustainable. Finally, implementation research (IR) involves studying how evidence-based practices can best be implemented in real-world settings outside of research contexts (eg, examining determinants of and strategies for implementation and evaluating and designing for the deployment context) [36]. In combination, CBPR stipulates principles for partnering with a community that may use a DMH program, HCD provides methods for effectively creating the DMH program such that end users will be engaged, and IR plans for and evaluates the deployment of the DMH program outside of a research setting. The frameworks improve the likelihood that DMH programs will have high uptake and a meaningful and sustained impact beyond research.

Across CBPR, HCD, and IR frameworks, the first step in creating interventions that are engaging and sustainable is to understand and center the needs, goals, and preferences of end users, their communities, and eventual deployment settings [31,33]. For example, previous research suggests that teens often experience anxiety through somatic symptoms and excessive worrying. However, it is unknown whether these findings fully generalize to the experiences of teen library patrons or whether there are additional factors to consider that are unique to the community they are in [37,38].

### Objectives

The purpose of this study was to use CBPR, HCD, and IR methods to understand the lived experiences of teen library patrons related to anxiety, elicit recommendations on what teen patrons would like to see in a DMH program for anxiety and anxiety management, and understand contextual factors and teens’ perspectives of the public library as a deployment setting. Our goal was to use this information to inform the adaptation of DMH programs that can be implemented in public libraries to meet teens where they already are and help alleviate the teen mental health crisis [15].

## Methods

### Setting and Participants

Building on our previous needs assessment with library workers [15], this study examined teen patrons’ needs and preferences related to the implementation of a DMH program for anxiety within a public library that serves communities in the west side and west of Chicago, Illinois. We will use *teens* or *teen patrons* to describe the teens who use the library frequently as this is the term the library community and teens use. The library serves community members from diverse racial and economic backgrounds, prioritizing the needs of marginalized patrons and those most affected by health inequities in their programming and the services they offer the community. AAK interviewed 16 teens aged 12-18 (mean 15.2, SD 2.0) years who used the library frequently at the time of the interviews. The interviewer held a master’s degree and doctoral degree in experimental psychology, was an assistant professor, and identified as female at the time of the interview. We define *frequently* as those teens who either attended weekly programming at the library or visited the library during the week. Of these 16 teen patrons, 56% (n=9) identified as female, 31% (n=5) identified as male, and 12% (n=2) identified as nonbinary, and 44% (n=7) identified as White individuals, 25% (n=4) identified as Black or African American individuals, 12% (n=2) identified as Hispanic or Latino individuals, 12% (n=2) identified with ≥2 races, and 6% (n=1) identified as Chinese American individuals.

### Procedures

All participants were recruited from a local Chicagoland public library between 2020 and 2021. The library’s Teen Services staff coordinated with the research team to post study flyers in the library common areas, promote the study in the library’s Teen Services programming, post in the library’s newsletter, and receive referrals from other teen patrons. Recruitment occurred until saturation was achieved in the interviews. No participant dropped out or refused to be part of the study; no repeat interviews were conducted. During the first year of the COVID-19 pandemic, the physical library was shut down; however, programming for teens continued via Zoom (audio only). Our research team built rapport with teens in person before the pandemic onset (and then once the physical library opened again), as well as attending the internet-based weekly programming events led by teens when the library was shut down. A total of 16 teen patrons participated in a 1-hour, individual needs assessment interview with members of the research study team.

Due to COVID-19 restrictions, all interviews were conducted over the phone or using audio-only Zoom calls (Zoom Video Communications). The semistructured interviews covered how participants experience and manage anxiety, current use of technology, and current or future library programming focused on teen mental health (Multimedia Appendix 1). Participants were also asked about what resources or features within a digital program may help them better manage anxiety. All interviews were audio recorded, transcribed, and uploaded to Dedoose (SocioCultural Research Consultants) [39] for qualitative data analysis. If teens expressed thoughts of suicide or self-harm behaviors during the interview, a predetermined safety procedure was in place to assess risk of imminent harm and take appropriate action (including safety planning for those deemed low risk and connecting the teens with emergency services for those at high risk). While some teens expressed experiencing self-harm thoughts in the past, no teen expressed current suicide or self-harm thoughts or behaviors, so the safety protocol was not enacted.

### Data Analysis

In total, 3 researchers (AAK, MH, and CW) conducted a thematic qualitative data analysis of the transcribed interviews. These coders all had training in qualitative analysis, all held master’s degrees, and one also held a doctoral degree. Coders used an iterative approach to create and refine the codebook. No field notes were used in the analysis. First, coders read and annotated 3 interview transcripts each, labeling relevant words, phrases, or sections with codes. Then, coders met to discuss and conceptualize the annotations, identify highly salient codes, and group codes into broader categories. The coders met regularly to discuss the application of codes, resolve discrepancies in code application, and refine the codebook accordingly. Next, coders were randomly assigned to apply the updated codebook to 5 or 6 additional interview transcripts each, after which they met to resolve discrepancies and further refine the codebook by removing codes that were underused or not present within the transcripts. After these iterations, the final codebook was applied to all transcripts, encompassing the following key themes: (1) public libraries and mental health, (2) teen anxiety experiences and management, and (3) DMH program design for teens and implementation considerations (see Multimedia Appendix 2 for the COREQ [Consolidated Criteria for Reporting Qualitative Research] checklist). The community members were primarily involved in member checking [40-43] in that they were most interested in reading the first drafts of the paper and verifying that the main concepts identified by researchers in the qualitative data analysis (and, thus, highlighted in the *Results* section) were an accurate representation of the teen patrons’ feedback and also of the library itself. The summary of the key themes is shown in Textbox 1.

**Summary of key themes and subthemes from the qualitative analysis conducted on the transcripts of the individual semistructured interviews with teens who frequently used a local Chicagoland library between 2020 and 2021. The interviews covered how teens experienced and managed anxiety; current use of technology; and current or future library programming focused on teen mental health, including digital mental health (DMH) programs for anxiety (Multimedia Appendix 1).**

**Public libraries and mental health**

- Most teens liked the centrality and easy access of the library.
- The library provides teens easy access to reliable mental health resources to connect with other teens on this subject.
- Teens from historically underserved racial and ethnic minority backgrounds and other marginalized identities perceived libraries as safe spaces.

**Teen lived experiences of anxiety**

- Uncontrollability: teens identified catastrophizing, overthinking, and a lack of control over thoughts, emotions, and actions as feelings of anxiousness. For some, anxiety led to feelings of sadness and depression and thoughts of self-harm. The cyclical pattern resulting from the co-occurrence of anxiety and depression symptoms severely impacted teens’ drive and motivation. The uncontrollability of anxiety led teens to feel burned out and tired, with a sense of dissociation from reality and derealization.
- Unpredictability: new experiences were a major source of anxiety related to unpredictability. Experiencing an unpredictable situation prompted urges to escape the situation or avoid future anxiety-provoking situations.
- Anger: anxiety can lead to teens getting easily frustrated, having a short fuse, and feeling angry. Sources of anger ranged from irritation over mistakes to frustration and bitterness toward personal anxiety. External annoyance and bitterness usually ended in arguments with family and friends.

**How teens manage anxiety**

- Breathing and progressive muscle relaxation: teens used apps with guided breathing, in-the-moment breathing exercises prompted by devices tracking physical changes, and progressive muscle relaxation to help regulate high anxiety levels.
- Grounding and distress tolerance: grounding techniques were used to disrupt uncomfortable thoughts and feelings. One method was the 5-4-3-2-1 technique, where one names things related to each sense. Another anxiety-managing technique used the sense of taste, namely, salty and sour tastes.
- Social connection: engaging in various activities with loved ones, namely, talking to and connecting with family and friends through conversation, helped with easing anxiety. Teens with anxiety felt validated and less isolated when sharing experiences with other teens or mental health professionals. Teens suggested an anonymous social component in a DMH program serving as a safe space for them to share experiences and advice with one another. Teens also wanted access to a mental health professional to provide advice in a safe space.
- Journaling and mood tracking: teens used journaling to work through and release their anxiety, helping them gain confidence and motivation to address stressors. Teens appreciated when mood tracking was used to reveal patterns such as relationships between their anxiety and daily activities. Some teens desired a pop-up mood tracker when first opening the program that could tailor mood management techniques to their input responses (eg, if high anxiety is reported, the program directs teens to in-the-moment anxiety relief techniques).
- Psychoeducation and crisis resources: teens found psychoeducation and crisis resources to be useful in contextualizing and learning the patterns and origins of their anxiety. Teens emphasized the importance of warm handoffs and easy access to reliable resources when in a crisis.
- Strategy choice and context: teens wanted a wide range of anxiety-mitigating techniques. Having variety allows teens to experiment and figure out what works best for them. Teens noted that context and anxiety levels played important roles in choosing a technique. The main goal of the resources should be to allow for practicing skills to eventually be used without technological assistance.

**Desired DMH program features**

- Personalization: teens liked app algorithms that curated or suggested content based on feedback, use, and data entry, as well as reports on patterns. They emphasized the need to understand who the program is targeting and provide resources specific to that population.
- Aesthetics and layout: teens preferred simple and concise app layouts that made navigation of the resources easy. Teens recommended that apps should be low commitment as longer materials were hard to keep up with and became overwhelming. They favored content delivered in teen-friendly language to keep the program more relatable and up-to-date.
- Gamification and animations: videos, animations and graphics, and mini games were the best modalities for information acquisition and engagement for teens. Content would be even more compelling if it featured teen celebrities or popular social media influencers. Teens liked interactive digital features, such as haptic and touch screen features, while practicing skills.
- Privacy and accessibility: protecting teens’ privacy was a top priority—teens worried about parental access to and surveillance of their phones and parents potentially finding out that they were struggling with mental health issues. Teens noted school rules and caregivers restricting phone use as access barriers. Paid subscriptions, an overt mental health focus in case of onlookers, and phone limits (eg, low storage and limited or inconsistent broadband access) were access barriers to resources on apps.

### Ethical Considerations

All research protocols were approved by the Northwestern University Institutional Review Board (STU00212314) before study enrollment. Both the interested teens and their caregivers provided permission to take part in the study. In the process of transcribing interviews, all potential identifying information was removed to protect the privacy of the participants, and each transcription was labeled as *P1*, *P2*, etc. Transcripts were not returned to participants. Each adolescent was given a US $25 Visa gift card for their time and participation in the interviews.

## Results

### Public Libraries and Teen Mental Health

Teens discussed resources they enjoyed at the library, the impact the library had on them, and their opinion on how libraries could support teen mental health. Most teens talked about the centrality and easy access of the library. P13 stated the following:

Because of where I get off at the train on the metro, I get off right across the street from the library so I can go to the library and just chill there.

Other teens described the library as a community access point. They noted that the library has many connections throughout the community and serves as a hub for resources.

When reflecting on the role public libraries should play in teen mental health, most teens agreed that the library is a great place for teens to access reliable mental health resources and connect with other teens about mental health via library programming. While some teens had specific ideas for new mental health offerings, such as creating a website or smartphone app to connect teens to resources to manage their mental health, they also spoke about resources that the library already had in place. For example, P8 described the following:

Like obviously books are a resource—and kind of just having that level of flexibility whether it’s mentorship or whether it’s grounding yourself in a story or whether it’s a pamphlet...having those different opportunities for how people have different or how different people interact with their own mental health and coping mechanisms.

Teens emphasized how the library was a safe space for them because of the trusted adults that worked there, friends they made at the library, and the library’s intentional teen programming. P12 summarized this nicely:

I was always at the library, and I was always doing these groups and these fun summer clubs. I had a lot of friends from the library...I do think that the library is a great resource place. There’s a lot of good, trusted adults. It’s quiet. And sometimes in a hectic household or a hectic friend fight, you might just wanna go to a place and feel welcomed and feel quiet and feel calm.

Another teen, P2, underscored their admiration for the trusted adults available via their teen programming at the library:

Even though you’re talking to an adult, you’re just talking to people you know that care about you.

The library was perceived as a safe space for many HURE and LGBTQIA+ teens in our sample, as well as those teens most experiencing inequities. Several of these teens mentioned that a social justice program within the library led by a local activist helped create a safe space in the library. In addition to weekly meetings for this program, teens researched and led town hall meetings educating the public on issues such as climate change’s disproportionate effect on lower-resourced communities. When asking P3 what they liked about this program, they responded the following:

For well me as like you know like a black, queer individual in every sense of the word, obviously because we [the group] love each other and stuff it’s great, but it’s also like I can just talk about this stuff...about stuff that I usually wouldn’t talk to normal people you know your average Joe or whatever about—talking about the oppression and the prison system and mass incarceration and all that stuff...But it definitely just helps so I can get all of those problems or frustrations out and also just for mental health just seeing these people [in the group]...It’s just like we’re a family and that’s really nice.

As can be seen in this quote, one HURE teen described finding solace in the shared experiences and similar backgrounds within this group, which allowed them to safely discuss topics including structural racism and oppression. Another teen, P13, described the following:

It’s just nice to have a space where I can be Black and annoyed and then there’s other people who get it.

Teens who attended this particular library program also emphasized their appreciation for the trusted adult who led the group, highlighting that the local activist empowered them and elevated their voices, challenging them to think critically while researching and reporting on current issues.

### Teens’ Lived Experiences of Anxiety

#### Overview

Teens shared about their experiences with anxiety, which impacted them in terms of physical sensations (eg, racing heart, difficulty breathing, and feeling shaky or flushed) and socially (eg, being easily embarrassed in public and worrying about disappointing or being judged or rejected by others). They also shared experiences less addressed in the teen anxiety literature; specifically, they spoke about their feelings of uncontrollability and unpredictability and the role of anger in their anxiety. As there is already a robust literature on physical sensations and social anxiety, we focused on these less explored aspects of teens’ experiences of anxiety.

#### Uncontrollability

Teens spoke about catastrophizing and overthinking as part of their anxiety, as well as feeling like their thoughts, emotions, and actions were out of their control. For example, P15 described the following:

My thought train is just going a million miles an hour. It’s like this thought leads to this other thought and it turns into a big fireball of negative thoughts...like out of control.

In addition to feeling a lack of control, some teens’ anxiety led to feelings of sadness and depression and thoughts of self-harm. P13 described their experience as follows:

For me, it’s usually like the anxiety comes and then the sadness once I cool down. So, I guess comparing it to a storm, it’s like the anxiety is the hurricane and then the sadness is kind of like the eye of the storm and then the anxiety comes back and then there’s a hurricane that follows it.

This teen highlighted the cyclical nature of symptoms, an experience shared by many others. Teens went on to describe how the co-occurrence of anxiety and symptoms of depression had a severe impact on their motivation, ultimately taking a toll on their grades and relationships with friends and family.

Finally, the uncontrollability of anxiety led some teens to feel burned out and tired, causing them to zone out, operate on autopilot, and shut down. They described feeling a sense of dissociation and derealization because of their uncontrollable anxiety. P4 described the following:

Dissociating and not feeling like I’m really present. Just kind of I become less aware of my surroundings.

They went on to describe an escalation in which they felt completely detached from reality.

#### Unpredictability

In addition to the uncontrollability of their anxiety, several teens spoke about the unpredictable nature of their anxiety, especially when it came to new experiences. For example, P7 explained the following:

If I go somewhere new, I think like, “Where is everything?” because I don’t know where anything is and then like, “What’s gonna happen? What am I gonna do? What are people gonna be like? Are people gonna judge me?”

Teens commonly described that experiencing an unpredictable situation led to urges to escape it or avoid future anxiety-provoking situations, such as new experiences. P9 described their reaction to new social situations:

I feel like people are staring at me. And all I wanna do is just hide usually. So, I try to get out of the situation, like wherever I am in public.

#### Anger

Several teens described getting easily frustrated, having a short fuse, and feeling very angry when anxious. Some internalized their anger, becoming irritated at themselves for making a mistake or experiencing frustration and bitterness toward their own anxiety and anxiety-provoking situations. Other teens described feeling annoyed and bitter externally, like they “needed to go punch something” (P16)*.* This usually ended in arguments with family and friends, adding to their anxiety and feelings of isolation.

### Teen Anxiety Management Strategies

#### Overview

Teens described using multiple strategies to manage their anxiety that included internally focused strategies for self-management as well as externally focused strategies for connecting with others and seeking resources. Teens reported diverse experiences with mental health treatment and resources—some described experiences with formal therapy, others used smartphone apps for mental health, some had experiences with both, and others had no previous experience engaging with these resources. We describe teens’ experiences in the following sections to inform future interventions that can promote and reinforce strategies that teens already feel comfortable using and that are already integrated into their daily lives. These methods of coping are well aligned with evidence-based strategies.

#### Breathing and Progressive Muscle Relaxation

Most teens mentioned that breathing exercises helped them manage anxiety in the moment. For example, P7 described using breathing exercises while having catastrophic and overwhelming thoughts:

Because I focus on my breathing instead of my thoughts. It kind of clears my mind too.

Teens also described using apps with guided breathing exercises (eg, facilitating box breathing and deep breathing) alongside interactive visualizations (eg, haptic and touch screen features). P6 described the following:

I really like the ones that are like circles that tell you to hold it in—that says to breathe in and hold it, then breathe out and hold it.

Other teens enjoyed apps that used inputs from other devices, such as smartwatches or exercise wristbands, to trigger breathing exercises when their heart rate increased. Other teens found utility in using breathing exercises as a supplemental strategy that could help once they had regulated their high anxiety levels using other strategies described in this section. Another anxiety-reducing technique that teens reported using in apps was progressive muscle relaxation. When describing a smartphone app that included this technique, P7 stated the following:

My favorite is where you tighten your muscles, which is like you ball your fists up and then relax them and stuff...I just kind of like it because it’s like you have all your stress and all your feelings and then you just let them go.

#### Grounding and Distress Tolerance

Several teens spoke about grounding and distress tolerance techniques, which were used to disrupt uncomfortable thoughts and feelings and then return to a more manageable level of anxiety. Some mentioned using the 5-4-3-2-1 technique when at their highest levels of anxiety—or, as P7 called it, “the five senses thingy”—where they named things related to each sense (eg, name 5 things they saw, 4 things they felt, and so on). When paired with breathing exercises, this technique made their anxiety manageable. Another teen, P6, described a unique technique:

When I feel really anxious, what I do is put salt on my tongue. So, that kinda just it distracts me from everything that I’m thinking about...It just disconnects me from reality for a bit because of the strong taste of salt messes up everything else that I was thinking about.

This teen mentioned doing the same exercise and achieving the same results with sour-tasting things such as lemons, limes, or vinegar.

#### Social Connection

All teens spoke about the importance of social connection in dealing with their anxiety even if social contexts brought on anxiety. P8 described this phenomenon as follows:

As much as I do have social anxiety, I’m a very social person.

The activities with friends and family that helped ease their anxiety ranged from low-key, in-person activities (eg, watching movies or listening to music together) to internet-based activities (eg, where teens would play video games together). The simple act of talking to and connecting with friends and loved ones was the most mentioned social activity that helped in easing anxiety, with teens emphasizing how these types of “conversations are really key in those [high-anxiety] moments” (P2). P8 described how conversations such as these helped:

Whether it’s just completely rambling you know like distracting me—or guiding me through breathing exercises or going on a walk with me and make sure I keep moving and that I’m paying attention to my surroundings like, “Oh, do you see that over there?” and that kind of thing and just helping to ground me and stuff.

Teens were asked to brainstorm how a DMH program could provide social connection to help with anxiety. Teens reflected that they felt validated and less isolated when reading about other teens’ experiences of anxiety and how they dealt with it. Accordingly, most teens suggested including an anonymous social component within a DMH program that would serve as “a safe space to talk to each other and see what people are going through” (P5). In addition to these shared experiences and advice, some teens wanted connection with a mental health professional or monitors to provide professional advice while also keeping it a safe space. P8 spoke to the benefits of this kind of platform:

Being able to ground yourself in other people’s experiences so that you can ground yourself in your own and feel more validated or feel making anxiety more approachable.

#### Journaling and Mood Tracking

Teens shared how journaling and mood tracking helped them alleviate or better understand their anxiety. P3 described how they used journaling to work through their anxiety:

After I’m done being upset in the moment, I’ll just write about that moment and it just kind of just helps me release those emotions—get them down so I remember them, remember what happened.

This cathartic nature of journaling was shared by most teens who used it as an anxiety management strategy. P14 had a unique technique where they would create characters and storylines based on a hardship or anxious situation they were experiencing. This teen garnered confidence and motivation to address stressors through these stories. P14 described how their story creation helped:

Yeah, it definitely takes me out of the moment and then it also it just feels like if I can beat this big milestone and do that and achieve that and then have it, homework’s nothing. I can finish that homework assignment because I can do this. I can do this. I’m more powerful than this homework. I will squash it, you know? Like a bug...It’s just a confidence thing.

Other teens saw value in reviewing patterns in their mood-tracking data, such as relationships between their anxiety and daily activities. P4 described the following:

It’s nice to see a daily tracker so you can see the bigger trend of what causes your stress and what periods and times you’re more stressed.

Teens desired a mood tracker that pops up when first opening the program so that their answers could tailor their interaction with the program. For example, if they entered a high anxiety rating, the program might direct the teens to in-the-moment anxiety relief techniques.

#### Psychoeducation and Crisis Resources

Several teens described the benefits of including psychoeducation and crisis resources in a DMH program embedded within the library. Teens found psychoeducation helpful in contextualizing and learning the patterns and origins of their anxiety. P1 expanded on their idea for integrating psychoeducation and resources into a DMH program:

I think it would be helpful to have a resources page...articles on anxiety, quick advice, I guess, and then maybe also a hotline page.

Teens also mentioned the importance of warm handoffs to reliable resources and the need for easy access to these resources when in a crisis. For example, P16 suggested that the future program should “definitely start off with, definitely a phone number to a social worker that if someone is feeling anxious, or they wanna hurt themselves, you can talk to them.”

#### Strategy Choice and Context

Overall, teens wanted a variety of anxiety-mitigating strategies to choose from as different strategies could work better or worse for different teens and as variety allows them to experiment to find what works best for them. Context and anxiety level played important roles in choosing a technique. For example, some teens described that they typically had their phones with them in public and would use social media or other apps to deal with their stress in the moment. P7 described the following:

It depends on where I am, but if I’m at home I’ll play my guitar, listen to music, or I’ll do meditation sometimes and sometimes I’ll draw...But if I’m in a public place, then I’ll do deep breathing.

Other teens expressed that their approach depended on how anxious they were, with some strategies (eg, physical activity) being better suited for high-anxiety moments and others (eg, psychoeducation and listening to music) being better suited for lower-anxiety moments. Regardless of the coping skill or how it was taught, the consensus from teens was that the main goal should be practicing skills to then use them without technological assistance when in-the-moment anxiety occurs. One participant described that “it’s not just about the technology or about the app” (P8). Most teens appreciated the supportive technologies they had previously used that helped them practice exercises, which in turn made it easier to use the acquired skills outside the app or in real life.

### Desired DMH Program Features

In addition to specific anxiety management strategies, teens discussed the top features they would like in a DMH program embedded within the library. These desired features fell under 4 broad themes: personalization, aesthetics and layout, gamification and animations, and privacy and accessibility.

#### Personalization

Teens discussed the importance of being able to both personalize and receive tailored information from programs. For example, P15 suggested the following:

You know how you can type something into WebMD and it will show what the problem is and also how to fix it. I think that’s what this app should look like. If you have anxiety over a bad grade, you can do like, “What can I do to overcome my anxiety about that or stress?”

As this example suggests, teens liked when apps used algorithms to curate and suggest tailored content based on their feedback, use, and entry of data into that app. Teens also appreciated reports on their particular use patterns (eg, features most used) and shortcuts to view these data. In addition, it was important that the mental health program offer resources specific to the population it was designed for. For example, P13 described the following:

One thing that I notice a lot with resources where people put resources for like, “Did you know about this and blah, blah, blah?” I’d be going through it, and it would be like, “This is clearly meant to educate white people.”

Several HURE teens underscored that current mental health resources were catered to a small group of people, White teens in particular, and did not reflect them or their mental health needs.

#### Aesthetics and Layout

Many teens preferred simple digital program layouts that were easy to navigate. They described being overwhelmed if the home screen of the program had too many options. For example, P11 said that they did not want the following:

A lot of folders or that you have to click a lot of things to get to something. I think it should be the important things, and then from there it can branch into more specific ways to cope with types of stress or recommendations or things like that.

Other teens emphasized that apps should be low commitment (ie, relatively quick and easy to navigate and use on a daily basis). Apps with higher commitment, or those that required significant time or energy to use, and those with longer materials were difficult to keep up with and quickly became overwhelming. Furthermore, teens shared that it would be important to have regularly updated content in teen-friendly language. P8 said the following:

Teens can be turned off by aesthetics that just feel kind of like dated or they feel like they were made by people much older than them.

#### Gamification and Animations

Several teens underscored that the best modalities for information acquisition and engagement were videos, animations and graphics, and mini games. P5 said the following:

Like a video lesson or something that the user could interact with or participate along with because there are many resources out there for a number of things...words don’t help as much as seeing the person or listening.

They went on to describe that using teen actors in videos could become cheesy but videos could be compelling if they featured teen celebrities or popular social media influencers. Teens also enjoyed interactive smartphone or tablet features (eg, haptic and touch screen features) where they could touch the screen while practicing skills. Teens gave examples that ranged from using the notification vibrations on the phone to guide deep breathing exercises to dragging their finger across the screen until an image was revealed.

#### Privacy and Accessibility

Protecting the teens’ privacy was among the top priorities. P6 emphasized the following:

Making sure that my information’s safe and only to me. It’s not shared with anyone else, especially, random people.

Other teens expressed worry regarding their parents’ access to and surveillance of their smartphones and them potentially finding out that they were struggling with mental health issues. P16, for example, stated the following:

It’s just my parents, every week, they checked my phone. And if they saw I was searching, “How to overcome anxiety,” I wouldn’t want them to make me the center of attention. And make them drop everything that they’re doing.

Some teens had their smartphone use restricted by caregivers or school rules. Policies ranged from not using smartphones during school hours to caregivers taking away teens’ phones as punishment (eg, when they said “something bad”) or at specific times (eg, being unable to use anything other than the music app on their smartphones after 8 PM on weeknights). Teens were also concerned about paid subscriptions, onlookers being able to see that the program was for mental health, and smartphone-specific barriers (eg, low battery, storage, and data of phone and inconsistent access to broadband). Many teens spoke about liking a particular app, such as a mood-tracking app, but reported that they stopped using it because they had to use their cellular data or pay to use the full version of the app, further limiting their use.

## Discussion

### Principal Findings

This study aimed to understand the needs and perspectives of teen library patrons in an effort to support the creation of a teen-focused DMH tool that can be disseminated in libraries. The results of the needs assessment interviews with teens who frequently use the library provided critical information on their perspectives of the library’s function and programming. Teens underscored the centrality of and, thus, easy access to their public library. They also referred to the library as a hub for connecting patrons to community resources and services. This is consistent with our previous research, in which library workers identified a main function of the library to be a conduit to guide patrons to desired resources [15].

Teens talked about the teen-specific programming they attended at their library, indicating that this is where they met a lot of their friends and fostered relationships with the trusted adults leading those programs. These factors led teens to view the library as a *safe space*, which is consistent with several other reports by library workers and researchers underscoring that communities see public libraries as safe spaces [27,28]. HURE teens and teens with marginalized identities (eg, LGBTQIA+ teens, teens living in lower-resourced and disinvested areas, and teens living with mental health conditions) found belongingness, companionship, and solidarity in library programming that spoke to different aspects of their identity. Given that there are more unsafe spaces for these groups due to marginalization, it is critically important to invest in and maintain safe spaces that bring individuals with shared experiences and similar backgrounds together and that celebrate the different aspects of their identities, especially within the teen population.

Teens shared the nuanced experiences they had regarding their anxiety. They spoke to the uncontrollable and unpredictable feelings associated with their anxiety, which led some teens to catastrophize, feel depressed, or have thoughts of self-harm, which then cyclically fed back into their anxiety. Other teens described dissociating to cope with adverse feelings or feeling the need to avoid or flee anxiety-provoking situations. There is evidence of the uncontrollable and unpredictable nature of anxiety in the adult anxiety literature, especially in relation to panic disorder, yet those variables have not been well studied in the teen anxiety literature and are worthy of future research [44-47]. More research is needed to better understand these 2 variables and their potential role as risk or maintenance factors, which could then be used as targets for DMH programs for anxiety and other mental health conditions in teens [48-52].

Other teens talked about their anger in relation to their anxiety. Some described the internal aspects of anger that may be expressed as frustration toward their own anxiety or frustration about making mistakes. Others described the external aspects of anger as experiencing frustrations that often led to conflicts with friends and loved ones. This finding is aligned with those of extant literature showing that anxiety and depression commonly manifest as anger and irritability in teens [4,48-53]. In addition, there is evidence in the literature suggesting that anger can be a prominent symptom co-occurring with several internalizing disorders [54]. Stress and anger as a mood symptom may be connected to experiences of racism among teens [54]. Similarly to other studies [54-56], our study shows that anger and racism-based stress play a role in the emotions of teens who may feel disenfranchised by the current status quo in society. Anger is often seen as a maladaptive response to stress; however, with regard to racism-triggered stress, anger can be an expected expression, and sometimes this expression allows for healthy processing of emotional stressors. Given that most of the teens in this study identified as HURE or multiracial, along with the literature indicating that anger is a known response to stress related to structural racism and discrimination, additional research is needed to better understand the relationship between anger (as a common stress response to racism) and anxiety among teens who are Black or Indigenous or hold other racial and ethnic identities. While the most potent intervention would be to address the ultimate problem of structural racism and discrimination, interventions that provide support and validation to HURE teens in the face of systemic racism and oppression are critical. For example, these programs could be offered as part of library services and provide teens with acknowledgment of factors such as privilege, oppression, and discrimination and ways to curate social safety, as well as coping strategies to alleviate the mood symptoms caused by racism [51,57,58].

Teens described a variety of coping strategies (eg, guided breathing, muscle relaxation, social connection, distress tolerance, and mood and behavior tracking) that they used to help feel more in control of their anxiety, lessen their symptoms, increase knowledge of anxiety patterns, and increase their motivation to approach rather than avoid anxiety-provoking experiences. For example, teens gained knowledge about their anxiety when mood tracking was used to reveal patterns such as relationships between their anxiety and daily activities. They liked this because it emphasized behaviors that they could change or control that led to observable effects on their mood. When applied to the design of a DMH program for teens, it may be beneficial to include some of these strategies as on-demand activities. Leveraging haptic and touch screen features to actively engage teens in these strategies, especially strategies that they are familiar with and are already integrated into their daily lives, may also be worthwhile [59,60].

In terms of overall goals of a DMH program, teens wanted to learn a variety of anxiety management strategies and, ultimately, be able to choose the most effective strategy in particular contexts (eg, exercise for high-anxiety moments, listening to music for low-anxiety moments, playing guitar at home, and deep breathing exercises in public). Interestingly, while they wanted the DMH program to facilitate learning of skills, they ultimately wanted to be able to practice these skills without technological assistance when in-the-moment anxiety occurred. This use goal is consistent with other reports from the DMH literature; for example, Latino and Latina [61] teens indicated that their main goals for using DMH programs were to practice skills within the program and then, ultimately, be able to use the learned skills *offline*. If teens are using DMH programs with this goal in mind, this could have implications for how we interpret engagement metrics with teen DMH programs [62]. Teens’ low, sporadic, or drop-off in engagement with a DMH program may not necessarily reflect a poor outcome or poor retention; rather, it could be that teens used the program as much as they needed to incorporate the skills learned into their daily lives. Mixed methods research is needed to better understand the patterns of teen engagement with DMH programs; how those patterns map onto teens’ actual use of the skills acquired in real life; and, ultimately, which patterns are most helpful in mental health management.

Teens shared their feedback on additional features that they would like to see in a DMH program for anxiety. It was important to teens that the aesthetics and function of the DMH program had the characteristics of clean design and easy navigation. Teens also stressed the importance of protecting their privacy, making the program accessible (eg, free and aligned with caregiver and school restrictions on devices), and designing discreet branding, all of which are consistent with several previous reports describing teens’ or therapists’ design recommendations regarding a teen DMH program [63,64]. In addition, teens reflected on the characteristics that increased their engagement with a digital program, which included gamification, videos and animations, and buy-in from teen celebrities or social media influencers. Other pediatric fields have found partnering with teen social media influencers to be a helpful strategy in enhancing knowledge and even outcomes of health care delivery [65]. In addition to the other features mentioned, partnering with popular teen social media influencers who are known for speaking about their mental health on social media outlets may be a promising strategy for increasing awareness and potentially intervention effectiveness of teen DMH programs. However, as teen culture and preferences rapidly and constantly shift (particularly regarding social media), DMH developers must balance ensuring relevance and timeliness of interventions with ensuring that the content still addresses theoretical mechanisms of change [66].

As for the additional feature of personalization, HURE teens underscored that the available teen mental health resources were White centric and they did not feel that *their* lived experiences were represented in current mental health resources. This suggests that we as scientists and designers need to intentionally improve the inclusion of populations who are underrepresented in teen mental health research, in particular HURE and LGBTQIA+ teens as well as those from lower-resourced and disinvested areas [67-76]. Teens advised that developers get to know the audience for the program and work with that population to personalize the program to their needs and preferences. This feedback raises an important design question: how do we equitably design DMH programs in community spaces that serve people with different identities as well as with different mental health needs and preferences? There are calls in the literature to move away from a *one-size-fits-all* model when developing DMH services [77-80]; however, more discussion and research are needed to better understand the best practices of DMH service development that is intended to meet the mental health needs and preferences of heterogeneous audiences (eg, a program encompassing tailored versions for subgroups within the heterogenous audience or different programs for different subpopulations) [81].

### Limitations

We would be remiss to not include a discussion on study limitations. The public library we worked with was well resourced with existing social services (eg, employed social workers, had a mental health and teen service budget, and offered free mental health assessments). Accordingly, the results of this study may not be generalizable to other public libraries that have different infrastructures and resources. More research is needed to better understand the teen resources offered by different public libraries and their branches across the United States. Second, we interviewed teen participants residing in a large, socially and politically progressive, and racially and ethnically diverse urban area. HURE teens living in areas with different sociopolitical environments and sociodemographic compositions may have different perspectives on whether and how they would like to engage in library-based DMH services. Third, we did not screen for anxiety before the interviews; however, it was apparent from teen interview responses that there was high variability in severity levels (level of interference) and treatment experience (some talked about their experiences with therapists, and some disclosed no experience with formal treatment). Finally, while the racial and ethnic makeup of the coders was diverse in representation, it is possible that our views as researchers led us to de-emphasize, overlook, or misinterpret aspects of the qualitative data, especially on topics such as equity, racism, and racial justice. The researchers took measures to reduce bias by engaging in researcher consensus throughout data analysis and member checking with teen and library staff from the partnering local library, some of whom are coauthors who reviewed and edited this report. We also included coauthors who are experts in the field of creating and testing DMH programs for Black, Indigenous, and other racial and ethnic minority populations.

### Conclusions

Disseminating and implementing DMH programs in public libraries is a promising strategy to increase access to care among teens in need of support for their anxiety. By understanding teens’ perspectives and designing tools with their needs centered, the promise of this approach may be realized. To the best of our knowledge, this is the first report elevating teen voices and opinions on implementing DMH programs in public libraries. Future research is now needed to test whether DMH programs adapted in accordance with the suggestions presented in this paper are acceptable, useful, and sustainable.

## Appendix

Multimedia Appendix 1 Interview script.

Multimedia Appendix 2 COREQ (Consolidated Criteria for Reporting Qualitative Research) checklist.

## Abbreviations

CBPR: community-based participatory research
COREQ: Consolidated Criteria for Reporting Qualitative Research
DMH: digital mental health
HCD: human-centered design
HURE: historically underserved racial and ethnic minority
IR: implementation research
LGBTQIA+: lesbian, gay, bisexual, transgender, queer, intersex, and asexual

## Data Availability

Due to the sensitive nature of the qualitative data with minors and the potential risk of identifying variables within the individual transcripts that would potentially impact the privacy of the sample, the individual interviews are not publicly available, nor will requests for data sharing be granted. In addition, public sharing of the individual interviews was not included in the teen assent and parent consent forms.
