# Supplementary material for: Teen Perspectives on Integrating Digital Mental Health Programs for Teens Into Public Libraries (“I Was Always at the Library”): Qualitative Interview Study
Source: JMIR Form Res. 2025 Mar 13;9:e67454. doi: 10.2196/67454 (PMC11950704; doi:10.2196/67454)
Supplement: Multimedia Appendix 1 [file formative_v9i1e67454_app1.docx]

**Supplemental Materials**

Design Opportunities for Mental Health Technologies for Teens

Semi-Structured One-on-One Telephone Interview

Defining and Managing Anxiety

People can have very different experiences when they feel anxious, stressed, or nervous. Some feel really uncomfortable, others feel their anxiety is like a really heavy weight on their shoulders. Some worry a lot about one specific thing, while others worry about more general things that could happen in the future. Some feel it in their bodies, they get sweaty and can’t catch their breath, or their heart starts to beat really fast. Others experience other kinds of emotions along with their anxiety, like feeling embarrassed or self-conscious in front of others. Some experience difficulties at school and with their friends and families, and most feel a combination of all of these things.

I am interested in how you experience anxiety and stress, and then what you usually do when feeling really anxious or stressed to work through those feelings. Please think of day in which you felt really anxious or stressed. If this is hard to remember, imagine the most recent time when you were feeling stressed or anxious.

Can you tell me about that day?

What was going on around you at the time?

What were you most stressed or worried about?

What kinds of thoughts, emotions, and bodily feelings did you have when you were stressed?

Which of these thoughts, feelings, or experiences were the worst and/or the hardest on you?

What is the biggest challenge in your life these days, that makes you feel anxious or stressed?

To feel less stressed or anxious, some people do activities like go outside, text with a friend, meditate, or listen to music. What do you do to help feel less anxious or stressed?

How do these things help you when you feel anxious or stressed?

Technology Use

Now, we are going to chat about technology. Think about the technology you use the most each day, such as a cell phone, computer, or tablet.

What technology do you use the most and what do you use it for?

What do you like most, or are your favorite features, of [most used technology] and why?

Have you used technology to make yourself feel better when you were really anxious or stressed? If so:

What technology did you use and how?

What about the [specific technology] helped with your stress?

Where did you use this technology (e.g., at school, at home, with friends or family)?

Have there been times when you are not allowed to use technology, like at school or at home (or as punishment), when it would have been helpful to deal with stress and anxiety?

I am also interested in learning why you might not use technology for stress and anxiety. For example, some people have said they have not used technology to help when anxious or stressed because they have spotty internet at home, or share devices with other family members, or because their cell phone is broken.

Are any of these true for you? If so, which ones?

What are other reasons, besides those we already talked about, why you have not used technology for anxiety or stress?

Let’s say you are going to design a smartphone app for teens to help with stress and anxiety. What would that app look like? What features would this app have?

If you could provide advice to a research team about to begin work with youth at the Oak Park Public Library to design a smartphone app, what would that advice be?
